# Draft Genome Sequence of *Archangium* sp. Strain Cb G35

**DOI:** 10.1128/genomeA.01678-16

**Published:** 2017-02-23

**Authors:** Barbara I. Adaikpoh, Scot E. Dowd, D. Cole Stevens

**Affiliations:** a Department of Biomolecular Sciences, University of Mississippi, Oxford, Mississippi, USA; b MR DNA, Molecular Research LP, Shallowater, Texas, USA

## Abstract

In an effort to explore myxobacterial natural product biosynthetic pathways, the draft genome sequence of *Archangium* sp. strain Cb G35 has been obtained. Analysis of the genome using antiSMASH predicts 49 natural product biosynthetic pathways. This genome will contribute to the investigation of myxobacterial secondary metabolite biosynthetic pathways.

## GENOME ANNOUNCEMENT

Myxobacteria produce a plethora of structurally diverse bioactive natural products (1–3). Isolated from tree bark in Bangalore, India, *Archangium* sp. strain Cb G35 (DSM 52696) was reported to produce the antibiotic roimatacene, as well as six novel *p*-hydroxyacetophenone amides (4, 5). Herein, we report a draft genome sequence for *Archangium* sp. strain Cb G35, which was collected in an effort to explore myxobacterial natural product biosynthesis.

*Archangium* sp. Cb G35 was acquired from the German Collection of Microorganisms (DSMZ) in Braunschweig (DSM 52696). *Archangium* sp. Cb G35 has been referenced as *Cystobacter ferrugineus* strain Cb G35 and *Cystobacter gracilis* strain Cb G35 prior to a suggested reclassification (4–6). Genomic DNA was isolated using a GeneJET genomic DNA purification kit (Thermo Fisher). Sequencing was performed at MR DNA (Shallowater, TX) using an Illumina HiSeq system. A Nextera DNA sample preparation kit was used for library construction (Illumina), according to the manufacturer’s user guide. Following the library preparation, the final concentration of the library (7.32 ng/μl) was measured using the Qubit double-stranded DNA (dsDNA) high-sensitivity (HS) assay kit (Life Technologies, Inc.), and the average library size (881 bp) was determined using the Agilent 2100 Bioanalyzer (Agilent Technologies). The libraries were pooled and diluted (to 10.0 pM) and paired-end sequenced for 500 cycles, with an average coverage of 50×. An initial annotation was completed using the Rapid Annotations using Subsystems Technology (RAST) server (7), with further annotation requested by the NCBI Prokaryotic Genome Annotation Pipeline (8, 9). The draft genome contains 12,927,638 bp with 89 identified RNAs, 10,395 coding sequences, and a 68.8% G+C content across 41 contigs containing protein-coding genes.

Ultimately, 49 unique secondary metabolite biosynthetic pathways were identified using antiSMASH (version 3.0.5), including pathways for 10 hybrid nonribosomal peptide polyketides, six nonribosomal peptides, six bacteriocins, five polyketides, five terpenes, four lantipeptides, and two microviridins (10). The biosynthetic pathways for reported natural products roimatacene and *p*-hydroxyacetophenone amides were not obvious from antiSMASH analysis and require further investigation. We believe the draft genome sequence will help facilitate the characterization of myxobacterial secondary metabolite biosynthetic pathways and the discovery of new myxobacterial natural products.

### Accession number(s).

This whole-genome shotgun project has been deposited in DDBJ/ENA/GenBank under the accession number MPOI00000000.
